# Determination of pharmacokinetics and tissue distribution of a novel lutetium-labeled PSMA-targeted ligand, ^177^Lu-DOTA-PSMA-GUL, in rats by using LC–MS/MS

**DOI:** 10.1038/s41598-022-19700-9

**Published:** 2022-09-14

**Authors:** Chang Ho Song, Kweon Kim, Eunhee Kang, Bora Jeong, Myung-Su Lee, Jiyoon Jung, Tae Hwan Kim, Soyoung Shin, Beom Soo Shin

**Affiliations:** 1 School of Pharmacy, Sungkyunkwan University, 2066 Seobu-ro, Jangan-gu, Suwon, Gyeonggi 16419 Republic of Korea; 2 CellBion Co., Ltd., Seoul, Korea; 3 College of Pharmacy, Daegu Catholic University, Gyeongsan, Korea; 4 College of Pharmacy, Wonkwang University, Iksan, Korea

**Keywords:** Drug development, Radiotherapy, Prostate cancer, Pharmaceutics, Mass spectrometry

## Abstract

Prostate specific membrane antigen (PSMA) is known to be overexpressed in prostate cancer cells, providing as a diagnostic and therapeutic target for prostate cancer. A lutetium-labeled PSMA targeted ligand, ^177^Lu-DOTA-PSMA-GUL is a novel radiopharmaceutical, which has been developed for the treatment of prostate cancer. While the GUL domain of ^177^Lu-DOTA-PSMA-GUL binds to the antigen, the beta-emitting radioisotope, ^177^Lu-labeled DOTA, interacts with prostate cancer cells. However, the in vivo pharmacokinetics of intact ^177^Lu-DOTA-PSMA-GUL has never been characterized. This study aimed to evaluate the pharmacokinetics and tissue distribution of the radiopharmaceutical in rats by using its stable isotope-labeled analog, ^175^Lu-DOTA-PSMA-GUL. A sensitive liquid chromatography-tandem mass spectrometry (LC–MS/MS) analysis of ^175^Lu-DOTA-PSMA-GUL was developed and validated. Following intravenous injection, the plasma concentration–time profiles of ^175^Lu-DOTA-PSMA-GUL showed a multi-exponential decline with the average elimination half-life of 0.30 to 0.33 h. Systemic exposure increased with the dose and renal excretion is the major elimination route. Tissue distribution of ^175^Lu-DOTA-PSMA-GUL was most substantial in kidneys, followed by the prostate. The developed LC–MS/MS assay and the in vivo pharmacokinetic data of ^175^Lu-DOTA-PSMA-GUL would provide helpful information for further clinical studies to be developed as a novel therapeutic agent for prostate cancer.

## Introduction

Prostate cancer is one of the most commonly occurring cancers worldwide. In 2020, there were more than 1.41 million new reported cases and approximately 375,000 deaths related to prostate cancer globally^1^. Although advances in the diagnosis and treatment have improved the ability to stratify patients by risk^2^, the mortality rate for prostate cancer has not been significantly reduced. In particular, as the incidence and mortality rates of prostate cancer are strongly related to age^3^, prostate cancer can threaten more people’s lives with the aging trend of the population^4^.

As an effective prostate cancer biomarker, prostate-specific membrane antigen (PSMA) has become an emerging diagnostic and therapeutic target. Originally found on the membrane of the prostate epithelium, PSMA is also detected in normal tissue cells like the prostate, kidneys, and small intestine^5^. However, PSMA is significantly overexpressed in prostate cancer cells compared to the level of antigen expression at other carcinoma tissues^6^. Moreover, PSMA overexpression is associated with higher prostate cancer grades and is of interest as a predictor of prostate cancer progression^7^. Thus, targeting agents for PSMA have been extensively investigated and developed for the diagnosis as well as treatment of prostate cancer^8–10^.

**Figure 1:**
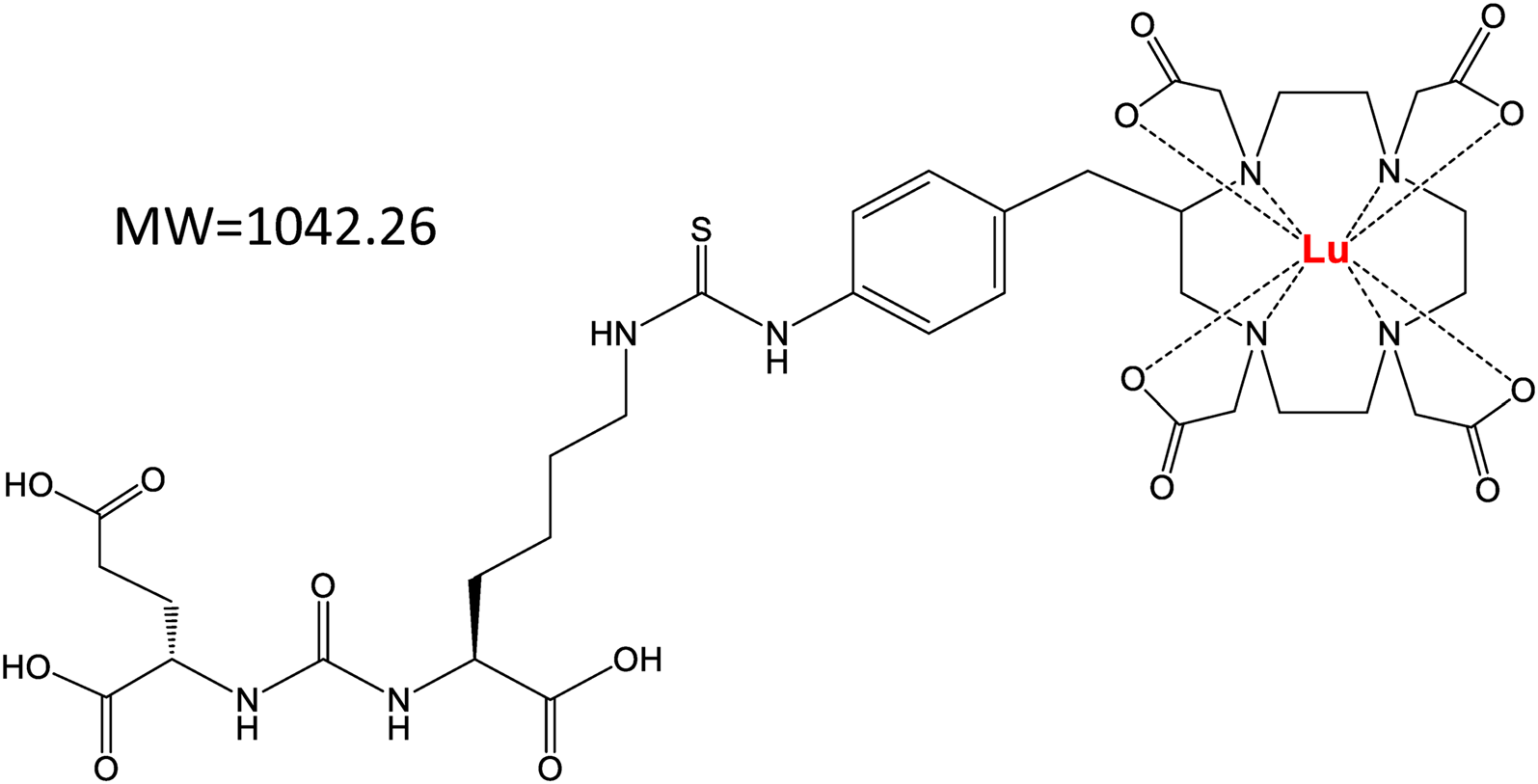
Structure of a Lu-labeled PSMA-targeted ligand, Lutetium labeled-1,4,7,10-tetraazacyclododecane-1,4,7,10-tetraacetic acid (DOTA)-PSMA-GUL.

Among various PSMA targeting agents, the urea-based PSMA ligand is one of the most advanced drug categories for imaging and therapy^10^. The urea-based PSMA ligands usually apply glutamate-urea-lysine (GUL) as a specific binding motif to PSMA. Representative GUL-based therapeutic PSMA ligands include ^131^I-MIP-1095, radionuclide-labeled PSMA-617, and PSMA-I&T. Particularly, ^177^Lu-PSMA-617 is one of the most studied radioligands currently under development^11^. With the high affinity for PSMA, ^177^Lu-PSMA-617 has shown promising results in the early clinical studies and demonstrated its activity in patients in recently completed phase 3 trials^9,12,13^. Radiotherapy with ^177^Lu-PSMA-617 also improved the overall survival and progression-free survival in mCRPC patients^14^. The safety and efficacy of ^177^Lu-PSMA-617 have also been confirmed by a long-term follow-up study^15^. In March 2022, ^177^Lu-PSMA-617 was finally approved by the FDA for the treatment of mCRPC. ^177^Lu-DOTA-PSMA-GUL is a structural analog of ^177^Lu-PSMA-617, developed to treat prostate cancer (Fig. 1). Both ^177^Lu-PSMA-617 and ^177^Lu-DOTA-PSMA-GUL have the GUL motif for specific binding to PSMA and the chelator 1,4,7,10-tetraazacyclododecane-1,4,7,10-tetraacetic acid (DOTA) complexed with a radionuclide, ^177^Lu, connected by a linker.

However, despite the extensive research interests and therapeutic potential, the in vivo pharmacokinetics and tissue distribution of the intact PSMA targeting ligands have not been fully characterized. The radioactivity study showed that ^177^Lu-PSMA-617 was initially distributed into extracellular body water and eliminated biexponentially with the elimination half-life of 90 h in humans^9^. High uptake and retention of radiation of these PSMA ligands in the tumors and PSMA expressing normal tissues have been observed^9,11,16–19^. However, these are dosimetry studies that estimate the radiation doses in target tissues or rely on imaging techniques such as PET/CT, rather than quantifying the intact ligands^9,11,16–19^. The major limitation in these radioactivity assays and imaging techniques is the inability to discern if the radionuclide is located in the intact drug or in the metabolites^20^. Thus, these methods are primarily indirect and likely affected by other factors. There are no reports related to the pharmacokinetic information on the radioligands or their analogs by quantitively determining its intact concentration in the biological samples. The direct and quantitative evaluation of the pharmacokinetics and tissue distribution has never been pursued for any PSMA ligands or analogs.

The main obstacle for complete pharmacokinetic characterization of the radiopharmaceuticals is mainly attributed to their inherent radioactivity. Radioactive compounds need to be treated only in dedicated spaces and facilities. Safety concerns also arise with the experiments with radioactivity, which makes it difficult to be combined with common analytical techniques like high-performance liquid chromatography (HPLC) or mass spectrometry. Particularly, sample pretreatment to extract the analyte from the biological samples for conventional analysis often requires elaborate steps and longer time. Thus, the pharmacokinetic information of radiopharmaceuticals relies on the easy alternative, i.e., radioactivity measurement. In the case of PSMA targeted ligands, their complex structures also contributed to the analytical difficulties. Although mass spectrometry-based^21^ and HPLC^22^ methods have been applied for quality control purposes, analytical methods that can accurately determine the concentration of PSMA targeted ligands in the biological fluids are not available.

Therefore, this study aimed to evaluate the pharmacokinetics and tissue distribution of the radiopharmaceutical ^177^Lu-DOTA-PSMA-GUL by using its radio-inactive analog, ^175^Lu-DOTA-PSMA-GUL in rats. A sensitive and robust liquid-chromatography tandem mass spectrometry (LC–MS/MS) for the quantification of ^175^Lu-DOTA-PSMA-GUL has been developed and validated in the biological matrices including plasma, urine, feces, and twelve different tissues to evaluate its pharmacokinetics and tissue distribution properties in rats. Stable isotopes combined with mass spectrometry have been effectively applied to determine pharmacokinetics or mode of action particularly when the conventional approach is not feasible^23^. This study might be the first report on the full characterization of the pharmacokinetics of an intact PSMA targeted ligand by applying mass spectrometry. The present LC–MS/MS analysis and the in vivo pharmacokinetic data of ^175^Lu-DOTA-PSMA-GUL may provide helpful information for the further development of Lu-labeled radiopharmaceuticals.

## Materials and methods

### Materials

1,4,7,10-tetraazacyclododecane-1,4,7,10-tetraacetic acid (DOTA)-PSMA-GUL (99.0%), 1,4,7-triazacyclononane-1,4,7-triacetic acid (NOTA)-PSMA-GUL (99.3%), and ^175^LuCl_3_ (99.9%) were obtained from CellBion (Seoul, Korea). Esomeprazole, sodium acetate, and acetic acid were purchased from Sigma Aldrich Chemical Co. (Milwaukee, WI, USA). Hydrochloric acid was obtained from Samchun Chemical Co., Ltd (Seoul, Korea). High-performance liquid chromatography (HPLC) grade acetonitrile, methanol, and water were purchased from J.T. Baker Co. (Philipsburg, NJ, USA).

### Preparation of ^175^Lu-DOTA-PSMA-GUL solution

The drug solution of ^175^Lu-DOTA-PSMA-GUL was prepared according to the method provided by Cellbion Co. (Seoul, Korea). Briefly, ^175^Lu-DOTA-PSMA-GUL was synthesized by mixing DOTA-PSMA-GUL solution and ^175^LuCl_3_ solution. DOTA-PSMA-GUL solution was prepared by dissolving 50 mg of DOTA-PSMA-GUL powder in 20 mL of 0.5 M sodium acetate buffer (pH 4.5). ^175^LuCl_3_ solution was prepared by dissolving 19 mg of ^175^LuCl_3_ in 10 mL of 0.04 M HCl. Then, 20 mL of DOTA-PSMA-GUL and 8.5 mL of ^175^LuCl_3_ were mixed for 20 min at 40 °C in a shaking water bath. The mixture was finally tested for the purity of ^175^Lu-DOTA-PSMA-GUL via HPLC–UV method using Waters 2695 separation module coupled with Waters 2487 dual wavelength absorbance detector (Waters, Milford, MA, USA). ^175^Lu-DOTA-PSMA-GUL was separated on an Agilent Zorbax 300SB-C18 (4.6 × 250 mm i.d., 5 μm, Agilent, Santa Clara, CA, USA) and detected at 244 nm.

### LC–MS/MS analysis condition

Liquid chromatography-tandem mass spectrometry (LC–MS/MS) analysis was performed by an Agilent 6490 triple-quadrupole mass spectrometer coupled with an Agilent 1260 HPLC (Agilent Technologies, Santa Clara, CA, USA). ^175^Lu-DOTA-PSMA-GUL in the rat biometrics (plasma, urine, feces, and 12 different tissue samples) was separated on an Agilent Zorbax SB-Aq column (100 × 2.1 mm, i.d., 3.5 μm, Agilent). Chromatographic separations were performed by using a binary gradient mobile phase composed of mobile phase A (1% formic acid in distilled water) and mobile phase B (1% formic acid in methanol). The gradient elution profile and flow rate was set as: 0 min, A:B = 95:5 (v/v), 0.3 mL/min; 8 min, 0:100, 0.3 mL/min; 10 min, 0:100, 0.3 mL/min; 10.01 min, 95:5, 0.5 mL/min; 15 min, 100:0, 0.5 mL/min; 15.01 min, 95:5, 0.3 mL/min; 22 min, 95:5, 0.3 mL/min. The gradient profile was optimized to improve the peak response and achieve rapid wash-out interference and equilibrate the column with the initial mobile phase condition for the next injection. The total run time was 22 min, and the column oven temperature was 40 °C. The sample injection volume was 5 μL.

The electrospray ionization (ESI) source was operated in positive mode, and the mass spectrometer was operated in the multiple reaction monitoring (MRM) mode. The observed MRM transitions and mass spectrometry settings are summarized in Supplementary Table 1.

### Preparation of stock solutions, calibration standards, and quality control samples

#### Stock solutions

The stock solutions of ^175^Lu-DOTA-PSMA-GUL were prepared by diluting 2.1 mg/mL synthesized solution in methanol to 400 μg/mL. The stock solutions of NOTA-PSMA-GUL (internal standard 1, IS1) and esomeprazole (internal standard 2, IS2) were prepared by separately dissolving 10 mg of each in 10 mL of methanol (1 mg/mL).

#### Calibration standards and quality control samples

For drug analysis in the plasma, calibration curves were constructed by spiking 50 μL of working stock solutions to blank plasma (50 μL each) to provide ^175^Lu-DOTA-PSMA-GUL concentrations at 20,000, 10,000, 5000, 1000, 500, 100, 50, and 20 ng/mL. The plasma was spiked with 50 μL of IS1 solution and 150 μL of methanol and mixed on a vortex mixer. The mixture was then centrifuged for 10 min at 4,000 rpm (3220 × g), and 100 μL of the supernatant was transferred to a plastic vial. After 100 μL of distilled water was added to the supernatant, the mixture was vortex-mixed for 10 min and 5 μL of the mixture was injected onto the LC–MS/MS. Quality control (QC) samples were prepared by spiking the working drug solutions to blank rat plasma to provide high concentration QC (16,000 ng/mL), middle concentration QC (8,000 ng/mL), low concentration QC (80 ng/mL) and lower limit of quantification (LLOQ) QC (20 ng/mL).

Similarly, calibration standards and QC samples were prepared for drug analysis in urine, feces, and twelve different tissues. Calibration ranges were 100–20,000 ng/mL for urine, 100–5000 ng/mL for feces and tissues. High, middle, and low QC sample concentrations were 16,000, 8000, and 400 ng/mL for urine, 4000, 1600, and 400 ng/mL for feces and tissue matrices.

### Sample preparation

For plasma samples, NOTA-PSMA-GUL (internal standard 1, IS1) solution 50 μL was added to 50 μL of the rat plasma. As a precipitation solvent, 200 μL of methanol was added, and the mixture was mixed on a vortex mixer for 10 min, followed by centrifugation for 10 min at 4000 rpm (3220 × g). After taking 100 μL of the supernatant, 100 μL of distilled water was added, vortex-mixed for 10 min. Finally, 5 μL of the prepared mixture was injected onto the LC–MS/MS. Since several plasma samples showed concentrations above the ULOQ, those samples were diluted 10- or 20-fold for analysis.

For urine and fecal homogenate samples, the working IS2 solution 50 μL was added to 50 μL of the homogenate samples. The samples were precipitated with methanol (900 μL) on a vortex mixer for 10 min, followed by centrifugation for 10 min at 4000 rpm (3220 × g). After taking 100 μL of the supernatant, 100 μL of distilled water was added, vortex-mixed for 10 min.

For tissue homogenate samples, the working IS2 solution 50 μL was added to 50 μL of tissue homogenate samples. The samples were precipitated with methanol (400 μL) on a vortex mixer for 10 min, followed by centrifugation for 10 min at 4,000 rpm (3220 × g). After taking 200 μL of the supernatant, 200 μL of distilled water was added to the supernatant and centrifuged for 10 min again. After the second centrifugation, 100 μL of supernatant and the same volume of distilled water was mixed for 10 min. Finally, 5 μL of the prepared mixture was injected onto the LC–MS/MS.

### In vivo pharmacokinetic studies in rats

#### Animals

Male Sprague–Dawley rats (7 weeks, 190–210 g; DBL co., Eumsung, Korea) were kept in plastic cages with free access to a standard diet (Youngbio, Seong-nam, Korea) and water. All experiments were performed in accordance with the relevant guidelines and regulations. The animal study protocol was approved by the Institutional Animal Care and Use Committee of Sungkyunkwan University (SKKUIACUC2018-07–25-1). Studies involving animals are reported in accordance with ARRIVE guidelines (https://arriveguidelines.org).

#### Pharmacokinetics of ^175^Lu-DOTA-PSMA-GUL after I.V. bolus injection

Freshly prepared ^175^Lu-DOTA-PSMA-GUL solution (2.1 mg/mL) was administered by i.v. bolus injection via the penile vein (n = 5–7) at three doses of 1, 2, and 5 mg/kg. Approximately 0.3 mL of the jugular venous blood samples were collected at predetermined times after i.v. injection. Plasma samples were harvested by centrifugation of the blood samples at 3220 × g for 10 min. Urine and feces samples were collected at 4, 8, 12, and 24 h after i.v. injection. All samples were stored at -70 °C until analysis.

#### Tissue distribution of ^175^Lu-DOTA-PSMA-GUL after I.V. infusion

Tissue distribution of ^175^Lu-DOTA-PSMA-GUL was examined under two different steady-state conditions after i.v. infusion of ^175^Lu-DOTA-PSMA-GUL. Two target steady-state plasma concentrations (C_ss_) were set as 3000 and 6000 ng/mL. Rats were surgically cannulated with polyethylene tubing (0.58 mm i.d. and 0.96 mm o.d.; Natume, Tokyo, Japan) in the left jugular vein for blood sampling and femoral vein for i.v. injection and infusion. After one day of recovery, ^175^Lu-DOTA-PSMA-GUL was administered by i.v. injection as a loading dose (LD) and i.v. infusion for 3 h to achieve the target C_ss_. The i.v. bolus LD and i.v. infusion rates (K_0_) were calculated by LD = C_ss,target_·V_ss_ and K_0_ = C_ss,target_·CL, respectively^24^. The volume of distribution (V_ss_ , 0.20 L/kg) and clearance (CL, 607.55 mL/h/kg) of ^175^Lu-DOTA-PSMA-GUL were obtained from the i.v. injection study. The calculated LD was 0.60 mg/kg and 1.20 mg/kg for the target C_ss_ of 3,000 ng/mL and 6,000 ng/mL, respectively. The calculated K_0_ was 1.82 mg/h/kg and 3.65 mg/h/kg for the target C_ss_ of 3,000 ng/mL and 6,000 ng/mL, respectively.

Blood samples were collected at 1.5, 2, 2.5, and 3 h during i.v. infusion, and centrifuged at 3,200 × g for 10 min. At the end of the infusion, rats were sacrificed, and brain, lung, heart, spleen, small intestine, stomach, kidney, liver, prostate, fat, muscle, and testis were excised and immediately homogenized in normal saline. All samples were stored at -70 °C until analysis.

#### Non-compartmental analysis

The plasma concentration–time data were analyzed by non-compartmental method using Phoenix^®^ WinNonlin^®^ (Pharsight, NC, USA). The fraction of ^175^Lu-DOTA-PSMA-GUL excreted into urine (F_urine_) and feces (F_feces_) were calculated by the ratio of the total amount of drug excreted in the urine and feces to the fraction of the dose, respectively. The tissue-to-plasma partition coefficients (K_P_) were calculated as the tissue-to-plasma concentration ratios.

#### Dose proportionality

Dose proportionality was tested for C_max_, AUC_all_, and AUC_inf_ based on power model. Assuming the natural logarithm of the pharmacokinetic parameter is linearly related to the natural logarithm of dose as in the following equation: ln(PK parameter) = β_0_ + β_1_ × ln(dose), the slope coefficient (β_1_) and its two-sided 95% confidence intervals (CI) were estimated.

### Statistical analysis

The data were statistically tested by the unpaired t-test to compare between two means and by one-way analysis of variance (ANOVA) followed by scheffe or games-howell post hoc test. The statistical significance level was set at p < 0.05. All the statistical analyses were performed by using IBM® SPSS® Statistics 26 (IBM, Armonk, NY, USA).

## Results

### Development of LC–MS/MS assay for ^175^Lu-DOTA-PSMA-GUL in rat biomatrices

The product ion mass spectra of protonated ^175^Lu-DOTA-PSMA-GUL, NOTA-PSMA-GUL (IS1), and esomeprazole (IS2) are shown in Supplementary Fig. 1. In the full scan of mass spectra, doubly charged ions, [M + 2H]^2+^ of ^175^Lu-DOTA-PSMA-GUL at m/z 522.1 and NOTA-PSMA-GUL were most intense, which was likely due to their complex structures. The most abundant product ions were observed at m/z 148 and 188.9 for ^175^Lu-DOTA-PSMA-GUL and NOTA-PSMA-GUL, respectively. Therefore, the MRM transitions of m/z 522.1 → 148.0 for ^175^Lu-DOTA-PSMA-GUL and m/z 385.8 → 188.9 for NOTA-PSMA-GUL were selected and monitored. For esomeprazole, the MRM transition of precursor to product ion pair was m/z 346.1 → 198.0.

The sample preparation and chromatographic conditions were optimized to increase the sensitivity of the analyte. The recovery was significantly enhanced when methanol was used as a precipitation solvent compared to acetonitrile. Thus, a single step protein precipitation with methanol was developed for the extraction of both analyte and internal standard (IS) from the biological samples. NOTA-PSMA-GUL was initially used as IS for the development of analytical method in the plasma. However, when the method was applied to other biological matrices, the recovery of NOTA-PSMA-GUL was poor (< 60%) and variable depending on the matrices. Thus, we have tried other compounds, including rebamipide, ketoprofen, diclofenac, aceclofenac, and esomeprazole, as an internal standard. Since esomeprazole showed higher recovery and acceptable reproducibility, it was selected as an internal standard for urine, feces, and tissue samples. For chromatographic separation, Agilent Zorbax SB-Aq column (100 × 2.1 mm, i.d., 3.5 μm) resulted in the best chromatographic separation with improved sensitivity after various reverse phase columns were examined. The presence of formic acid in the mobile phase significantly increased the peak response. The use of methanol as an organic solvent was also beneficial compared to acetonitrile. Finally, the gradient profile was optimized to provide the best sensitivity of ^175^Lu-DOTA-PSMA-GUL with the minimal interference.

### Validation of LC–MS/MS assay

**Figure 2:**
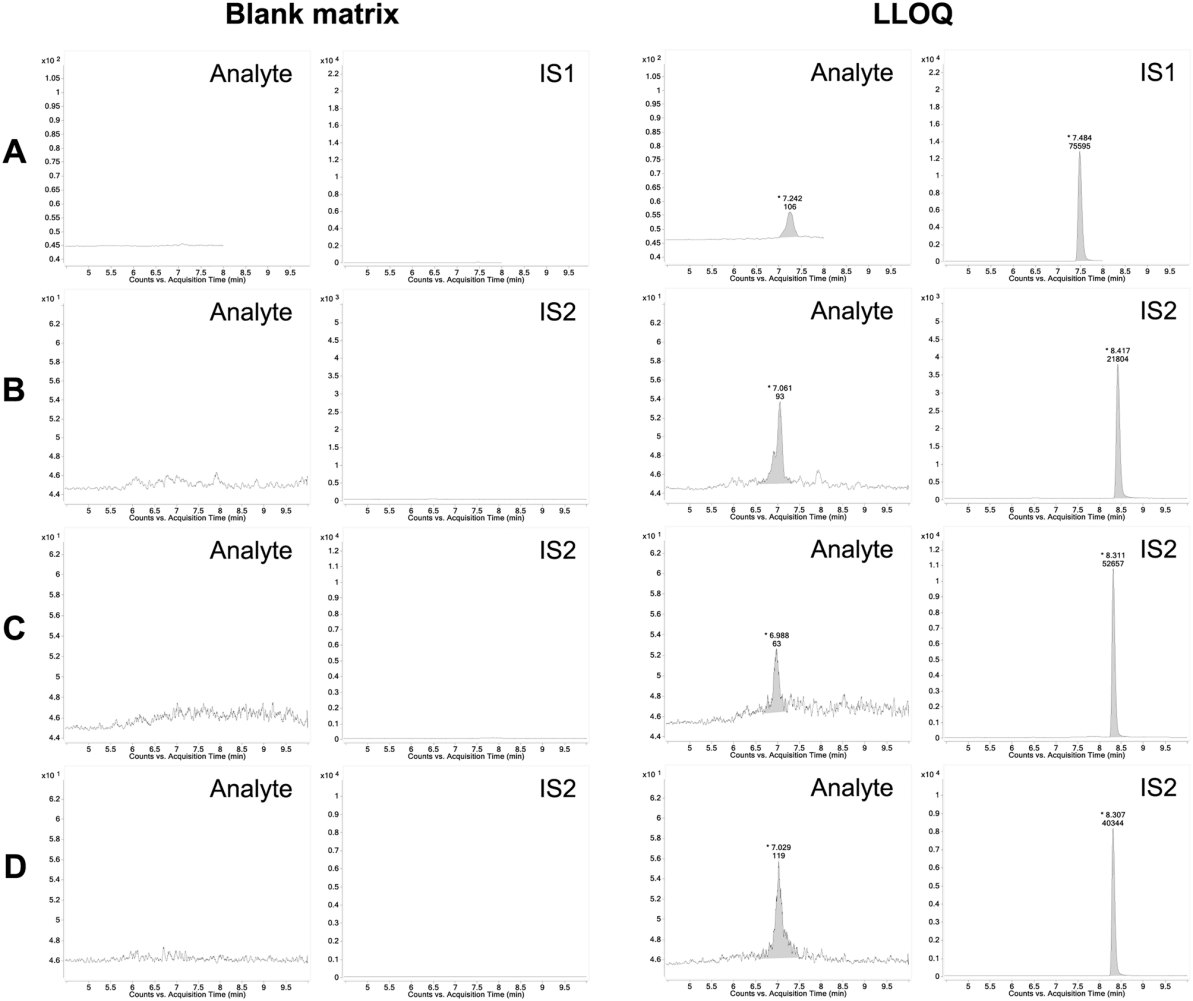
Representative MRM chromatograms of ^175^Lu-DOTA-PSMA-GUL (analyte) and internal standard (IS) obtained from blank matrix (left) and blank matrix spiked with ^175^Lu-DOTA-PSMA-GUL at LLOQ and IS (right) for plasma (**A**), urine (**B**), feces (**C**), and prostate (**D**).

The LC–MS/MS assay was validated according to the FDA guidance^25^. Representative MRM chromatograms of ^175^Lu-DOTA-PSMA-GUL and IS are shown in Fig. 2. Examination of blank sample, zero sample and other calibrators showed no interfering peaks at the retention times corresponding to the analyte and internal standards in all matrices, indicating the specificity of the assay. The calibration curves were linear over ^175^Lu-DOTA-PSMA-GUL concentration ranges of 20–20,000 ng/mL for plasma, 100–20,000 ng/mL for urine, and 100–5,000 ng/mL for feces and all tissues with R^2^ > 0.998. The lower limit of quantification (LLOQ) was 20 ng/mL in the plasma and 100 ng/mL in other biological matrices, including urine, feces, and tissue samples. The signal-to-noise (S/N) ratios at LLOQ concentration were over 11.7 for all tested biological matrices.

The intra- and inter-day accuracy and precision determined by assaying LLOQ, low, medium, and high QC samples in the plasma, urine, feces, and twelve tissues are shown in Supplementary Table 2. The intra- and inter-day accuracy ranged from 90.96 to 106.82% in all the rat biological matrices. The intra- and inter-day precisions were under 10.22% and 9.22%, respectively. The intra-day and inter-day accuracy and precision were all within the ranges recommended by the FDA^25^.

Analytical validation data including stability and process efficiency are shown in Supplementary Tables 3 and 4. Compared with the theoretical concentrations, ^175^Lu-DOTA-PSMA-GUL concentrations in each biological matrix did not deviate significantly after storage under four different conditions including short-term (room temperature for 4 h), long-term (-70 °C for two weeks), freeze–thaw (4 cycles), and autosampler (4 °C for 24 h) (Supplementary Table 3). These data indicates that ^175^Lu-DOTA-PSMA-GUL was stable in each biological matrix under the sample collection and assay conditions. The process efficiency in each biological matrix is generally consistent (Supplementary Table 4). Although the process efficiency of ^175^Lu-DOTA-PSMA-GUL was found to be lower in several tissues such as lung, small intestine, kidney, and liver, they were consistent and reproducible as indicated by the small CV%. FDA guidance also indicated that “Recovery need not be 100 percent, but the extent of the recovery of an analyte and of the ISs should be consistent and reproducible.”^25^ For plasma samples, the dilution integrity was evaluated for a 10- and 20-fold dilution of QC samples at 40,000 ng/mL, which was two times higher than ULOQ, with blank matrix. The accuracy for dilution integrity was 105.56% and 102.21%, while precision was 1.03% and 2.39% for tenfold and 20-fold dilution, respectively (n = 3, each).

### Pharmacokinetics of ^175^Lu-DOTA-PSMA-GUL in rats

A drug solution of ^175^Lu-DOTA-PSMA-GUL was prepared immediately before drug administration and tested by HPLC–UV. The purity of ^175^Lu-DOTA-PSMA-GUL was > 95%. The peak response of ^175^Lu-DOTA-PSMA-GUL was consistent (97.1 ± 8.0%) compared to the reference, indicating that ^175^Lu-DOTA-PSMA-GUL was uniformly prepared.

**Figure 3:**
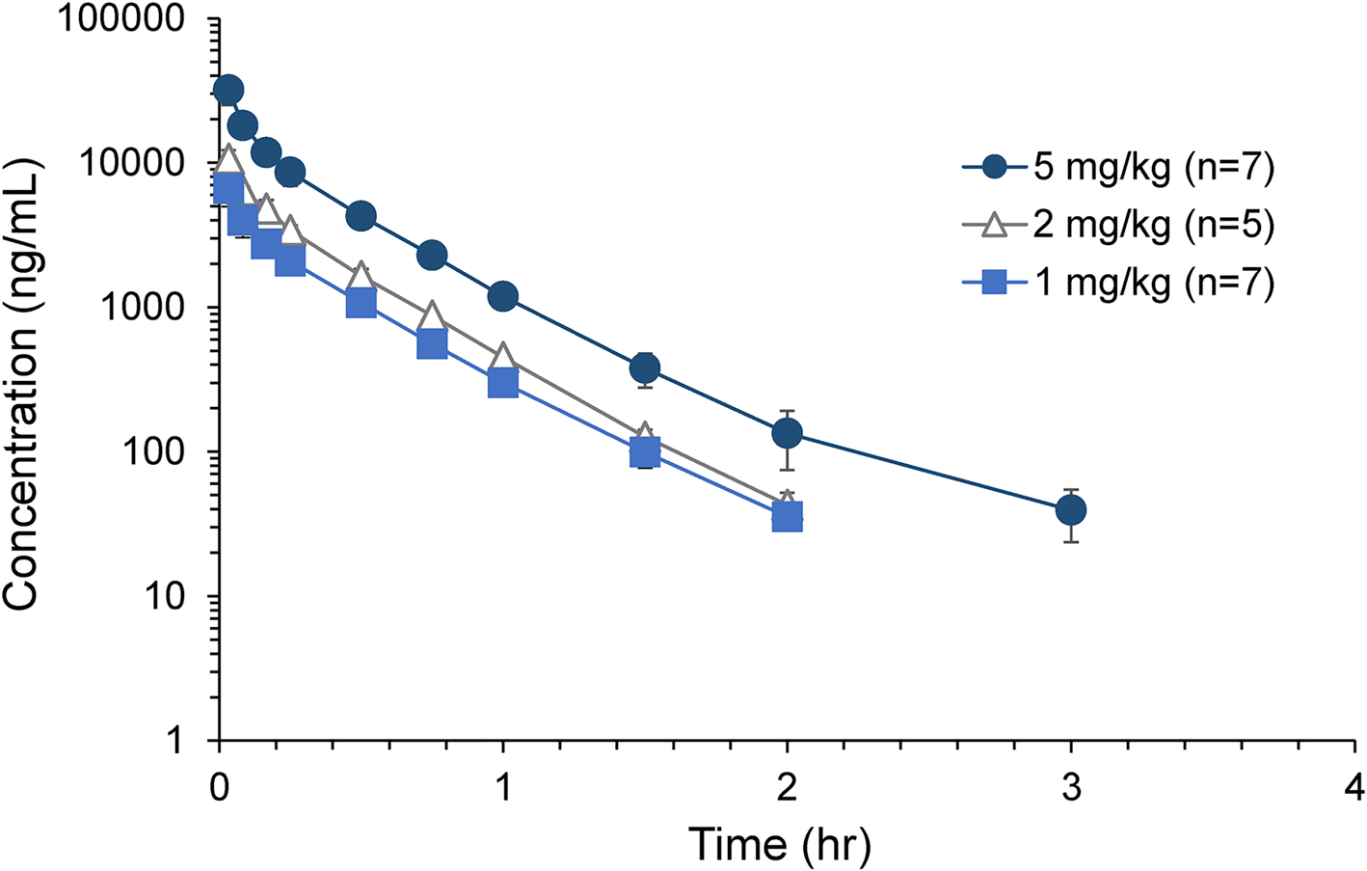
Average plasma concentration of ^175^Lu-DOTA-PSMA-GUL vs. time profiles obtained after intravenous injection of 1, 2, and 5 mg/kg ^175^Lu-DOTA-PSMA-GUL in rats (n = 5–7).

Figure 3 shows the average plasma concentration of ^175^Lu-DOTA-PSMA-GUL vs. time profiles after a single i.v. bolus injection of ^175^Lu-DOTA-PSMA-GUL (1, 2, and 5 mg/kg). The non-compartmental pharmacokinetic parameters of ^175^Lu-DOTA-PSMA-GUL are presented in Table 1. As shown in Fig. 3, the plasma concentration of ^175^Lu-DOTA-PSMA-GUL showed multi-exponential decline following i.v. injection in rats. The average elimination half-life of ^175^Lu-DOTA-PSMA-GUL was less than 0.33 h, and ^175^Lu-DOTA-PSMA-GUL was not detected in the plasma after 4 h of i.v. injection. The average CL and V_ss_ of ^175^Lu-DOTA-PSMA-GUL were estimated to be 10.13 mL/min/kg and 0.20 L/kg, respectively.

**Table 1:** Average non-compartmental pharmacokinetic parameters and excretion ratio of ^175^Lu-DOTA-PSMA-GUL after intravenous injection in rats (n = 5–7).

Parameters	1 mg/kg	2 mg/kg	5 mg/kg
t_1/2_ (h)	0.32 ± 0.02	0.30 ± 0.03	0.33 ± 0.09
C_0_ (ng/mL)	9267.42 ± 2673.59	14,960.22 ± 3104.40*	46,720.92 ± 13,829.91*^†^
AUC_all_ (ng·h/mL)	1841.19 ± 266.36	3029.00 ± 350.80*	8038.10 ± 1055.28*^†^
AUC_inf_ (ng·h/mL)	1857.48 ± 270.08	3047.45 ± 346.00*	8076.44 ± 1063.99*^†^
V_z_ (L/kg)	0.25 ± 0.03	0.29 ± 0.05	0.30 ± 0.09
CL (mL/min/kg)	9.13 ± 1.30	11.04 ± 1.15	10.46 ± 1.30
MRT (h)	0.34 ± 0.02	0.32 ± 0.03	0.32 ± 0.02
V_SS_ (L/kg)	0.19 ± 0.03	0.21 ± 0.04	0.20 ± 0.03
F_urine_ (%)	58.71 ± 21.26	54.59 ± 25.98	59.89 ± 22.82
F_feces_ (%)	5.46 ± 4.11	1.08 ± 0.76	6.41 ± 3.68

*p < 0.05 vs. 1 mg/kg; ^†^p < 0.05 vs. 2 mg/kg.

#### Dose proportionality

The plasma concentration at time zero (C_0_), the area under the plasma concentration–time curve from time zero to last time point (AUC_all_), and AUC from time zero to infinity (AUC_inf_) increased with the increase of ^175^Lu-DOTA-PSMA-GUL dose. Assessment of dose proportionality by the power model showed that the slope estimates (β_1_) were 1.012 [95% CI 0.830–1.194] for C_0_, 0.923 [95% CI 0.825–1.021) for AUC_all_, and 0.921 [95% CI 0.822–1.019] for AUC_inf_. Thus, the dose proportionality was statistically significant for all tested systemic exposure parameters, i.e., C_0_, AUC_all_, and AUC_inf_, indicating the linear pharmacokinetics of ^175^Lu-DOTA-PSMA-GUL over i.v. dose range from 1 to 5 mg/kg.

#### Excretion of ^175^Lu-DOTA-PSMA-GUL

The excretion of ^175^Lu-DOTA-PSMA-GUL via urine and feces was determined by analyzing ^175^Lu-DOTA-PSMA-GUL concentrations in the urine and feces samples for 24 h after i.v. injection. The fraction of ^175^Lu-DOTA-PSMA-GUL excreted into urine (F_urine_) and feces (F_feces_) are also shown in Table 1. The average F_urine_ of ^175^Lu-DOTA-PSMA-GUL was 54.59–59.89%, indicating that renal excretion may be the major elimination pathway of ^175^Lu-DOTA-PSMA-GUL. On the other hand, the average F_feces_ of ^175^Lu-DOTA-PSMA-GUL was 1.08–6.41%, indicating the marginal excretion of into feces.

### Tissue distribution of ^175^Lu-DOTA-PSMA-GUL

#### Steady state

Tissue distribution of ^175^Lu-DOTA-PSMA-GUL was assessed at steady state following i.v. infusion at two dose levels that aimed to obtain C_ss,target_ of 3,000 ng/mL and 6,000 ng/mL. An i.v. bolus loading dose was also administered to rapidly achieve the target steady state concentration.

**Figure 4:**
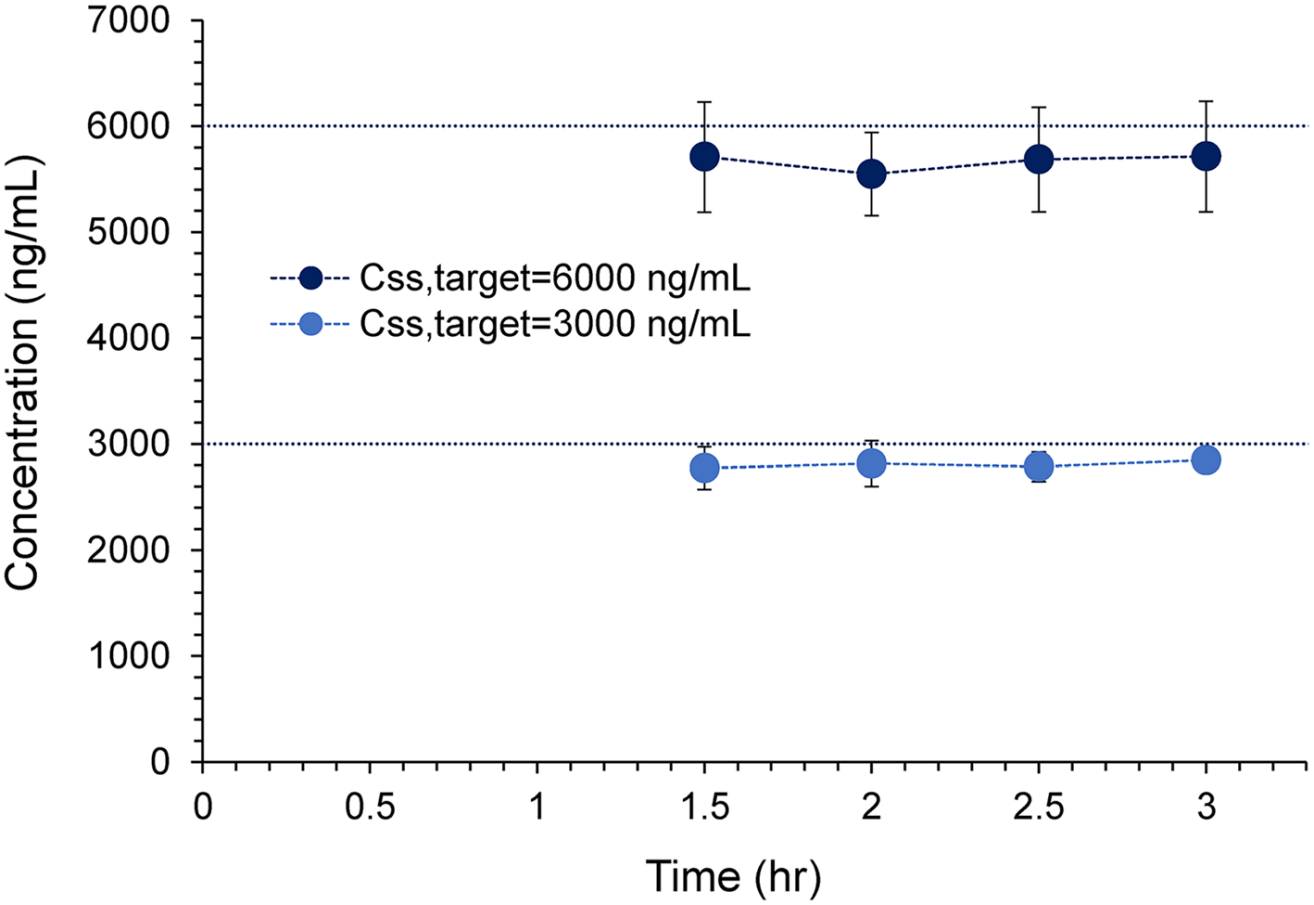
Average plasma ^175^Lu-DOTA-PSMA-GUL concentration vs. time curves obtained following continuous i.v. infusion (K_0_ = 1.82 and 3.65 mg/h/kg) with i.v. bolus loading dose (LD = 0.60 and 1.20 mg/kg) in rats (n = 5, each).

Average plasma ^175^Lu-DOTA-PSMA-GUL concentrations determined following simultaneous i.v. bolus injection (loading bolus dose 0.60 and 1.20 mg/kg) and continuous i.v. infusion (infusion rate 1.82 and 3.65 mg/h/kg) are shown in Fig. 4. Plasma concentrations of ^175^Lu-DOTA-PSMA-GUL appeared stable at approximately 3,000 ng/mL and 6,000 ng/mL from 1.5 h following i.v. infusion. These data indicate that the steady-state has been reached, and the intended steady-state plasma concentrations have been achieved.

#### Tissue distribution

**Figure 5:**
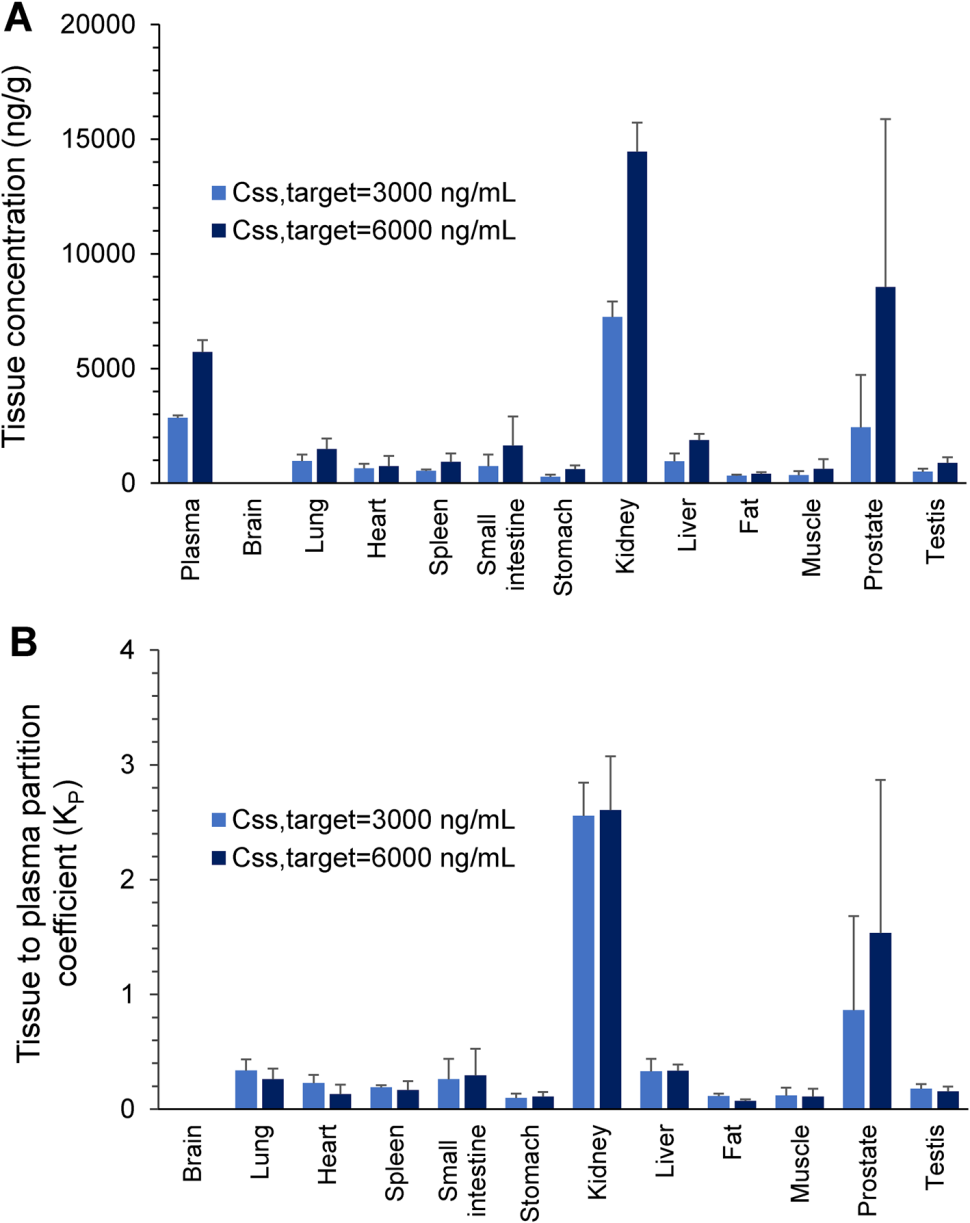
Average tissue concentrations (A) and tissue-to-plasma partition coefficients (K_P_) of ^175^Lu-DOTA-PSMA-GUL (B) obtained following continuous i.v. infusion (K_0_ = 1.82 and 3.65 mg/h/kg) with i.v. bolus loading dose (LD = 0.60 and 1.20 mg/kg) in rats (n = 5, each).

After confirming that the steady state has been achieved, tissue samples were collected to assess the tissue distribution of ^175^Lu-DOTA-PSMA-GUL. The average tissue concentrations of ^175^Lu-DOTA-PSMA-GUL and the respective K_P_ in twelve different tissues at steady state are shown in Fig. 5 and Table 2. The tissue concentrations are presented in ng/g tissue, which was calculated as the measured concentration in the tissue homogenate by LC–MS/MS (ng/mL) divided by the density of tissue homogenate (g tissue/mL). Tissue concentration of ^175^Lu-DOTA-PSMA-GUL was the highest in the kidneys, followed by the prostate, lung, liver, small intestine, heart, spleen, testis, muscle, fat, and stomach. ^175^Lu-DOTA-PSMA-GUL was not detected in the brain. Accordingly, the average K_P_ values of kidneys and prostate were 2.58 and 1.20, which are greater than 1.0 and significantly higher than those of other tissues, indicating that ^175^Lu-DOTA-PSMA-GUL is highly distributed into these tissues. The average K_P_ for other tissues was less than 0.34.

**Table 2:** Average concentrations of ^175^Lu-DOTA-PSMA-GUL and tissue to plasma partition coefficient (K_P_) in tissues obtained following simultaneous intravenous bolus injection (loading bolus dose 0.60 and 1.20 mg/kg) plus continuous intravenous infusion (infusion rate 1.82 and 3.65 mg/h/kg) in rats (n = 5, each).

Tissues	Concentration (ng/g)		Tissue to plasma coefficient (K_P_)	
	C_SS,target_ = 3000 ng/mL	C_SS,target_ = 6000 ng/mL	C_SS,target_ = 3000 ng/mL	C_SS,target_ = 6000 ng/mL
Plasma*	2845.62 ± 111.43	5713.2 ± 523.23	–	–
Brain	BLOQ	BLOQ	–	–
Lung	962.76 ± 287.82	1480.52 ± 464.64	0.34 ± 0.10	0.26 ± 0.09
Heart	645.67 ± 204.29	737.57 ± 453.10	0.23 ± 0.07	0.13 ± 0.08
Spleen	541.13 ± 52.33	932.03 ± 368.50	0.19 ± 0.02	0.17 ± 0.08
Small intestine	741.67 ± 507.55	1636.21 ± 1273.92	0.26 ± 0.18	0.29 ± 0.23
Stomach	276.46 ± 95.71	612.39 ± 161.18	0.10 ± 0.04	0.11 ± 0.04
Kidney	7253.09 ± 665.80	14,455.60 ± 1272.17	2.56 ± 0.29	2.61 ± 0.47
Liver	949.81 ± 342.61	1881.97 ± 267.48	0.33 ± 0.11	0.34 ± 0.05
Fat	320.68 ± 55.09	406.59 ± 77.17	0.11 ± 0.02	0.07 ± 0.02
Muscle	342.27 ± 188.70	621.78 ± 419.81	0.12 ± 0.07	0.11 ± 0.07
Prostate	2433.69 ± 2292.84	8550.82 ± 7322.84	0.86 ± 0.82	1.54 ± 1.33
Testis	504.17 ± 122.39	877.83 ± 252.52	0.18 ± 0.04	0.15 ± 0.04

*Plasma concentrations are in ng/mL; BLOQ, below the lower limit of quantification.

## Discussion

This study aimed to evaluate the pharmacokinetics of an intact PSMA-targeted ligand in rats. The introduction of an isotopically labeled compound combined with mass spectrometry allowed highly sensitive and specific bioanalysis as well as a full characterization of the pharmacokinetics.

A robust quantification method of ^175^Lu-DOTA-PSMA-GUL has been developed using LC–MS/MS to analyze the intact ^175^Lu-DOTA-PSMA-GUL in the biological fluids, including plasma, excreta, and 12 different tissues. Although sensitive mass spectrometry-based analytical methods have become essential for any pharmacokinetic studies for radio-inactive compounds, there have been limited attempts to actively utilize LC–MS/MS for radioactive ligands. Conventional analytical methods such as LC–MS/MS for the intact compound might be either infeasible due to the radioactivity of the radioligands or not demanding due to the presence of alternatives such as radioactivity and imaging techniques. Nevertheless, the radioactivity assays and imaging techniques are primarily indirect and may result in inaccurate results because they cannot discern the radionuclide located in the intact drug or in the metabolites^20^. Therefore, we utilized a stable isotope-labeled analog, ^175^Lu-DOTA-PSMA-GUL, combined with LC–MS/MS to characterize the pharmacokinetic behavior of a PSMA-targeted radioligand, ^177^Lu-DOTA-PSMA-GUL.

By using the LC–MS/MS, the pharmacokinetic profiles and excretion of ^175^Lu-DOTA-PSMA-GUL were first examined after i.v. bolus injection in rats. Following i.v. injection, ^175^Lu-DOTA-PSMA-GUL exhibited multi-exponential decline with a relatively short elimination half-life (t_1/2_) of 0.30–0.33 h. The rapid clearance from the systemic circulation of PSMA ligands has already been recognized^10,26^. Our data also showed that the systemic exposure represented by C_0_, AUC_all_, and AUC_inf_ was dose-proportional over the doses of ^177^Lu-DOTA-PSMA-GUL from 1 to 5 mg/kg. Over 50% of the administered ^175^Lu-DOTA-PSMA-GUL was excreted into the urine, suggesting that renal excretion is the primary elimination route of ^175^Lu-DOTA-PSMA-GUL, whereas the excretion into feces was insignificant. These results are in good agreement with the clinical reports that the majority of ^177^Lu-PSMA-617 was eliminated by urine (approximately 50%) and only 1–5% by fecal excretion in patients with PSMA-positive tumor phenotype^9^.

The distribution of ^175^Lu-DOTA-PSMA-GUL was assessed at two steady state conditions after intravenous infusions of ^175^Lu-DOTA-PSMA-GUL in rats. Although PSMA targeted radioligands are reported to be highly distributed to PSMA expressing tumors and normal organs like kidneys via imaging technology^9,11,16–19^, direct experimental evidence of their distribution into various tissues has not been reported. Moreover, in the present study, the in vivo tissue distribution of ^175^Lu-DOTA-PSMA-GUL has been determined at steady states (Fig. 4). At steady state, the rate of drug input, i.e., intravenous infusion, is equal to the rate of drug output, i.e., elimination, resulting in no net change in the amount of drug in the body. Thus, steady state conditions allow evaluating the true extent of tissue distribution when the plasma and tissue concentration do not change, which may be affected by confounding factors otherwise.

From the plasma concentration–time data obtained after i.v. injection, the average volume of distribution (V_ss_) was estimated to be low, i.e., 0.20 L/kg (Table 1), indicating that the distribution of ^175^Lu-DOTA-PSMA-GUL into tissues may be limited. Indeed, the observed concentrations of ^175^Lu-DOTA-PSMA-GUL in all tested tissues except the kidneys and prostate were lower than half of the plasma concentration (Table 2). The limited tissue distribution of ^175^Lu-DOTA-PSMA-GUL is likely due to its large molecular weight, preventing the passage across the cell membrane. Nevertheless, our data clearly showed that the distribution of ^175^Lu-DOTA-PSMA-GUL into the kidneys and prostate was significantly higher than other tissues (Table 2 and Fig. 5). The kidneys and prostate are the well-known tissues where PSMA is expressed^5^. The high distribution in the kidneys may also be attributed to that the primary elimination route of ^175^Lu-DOTA-PSMA-GUL is urinary excretion. As presented in Table 1, more than half of the ^175^Lu-DOTA-PSMA-GUL is excreted into the urine. On the other hand, the high K_P_ of ^175^Lu-DOTA-PSMA-GUL in the prostate may be mainly associated with the prostate targeting property of ^175^Lu-DOTA-PSMA-GUL. The GUL domain of ^175^Lu-DOTA-PSMA-GUL binds explicitly to PSMA that is overexpressed in the prostate cancer cells, providing the prostate targeting property^10^. These tissue distribution data indicate that ^177^Lu-DOTA-PSMA-GUL may be specific and effective for the treatment of prostate cancer.

In summary, the pharmacokinetics of a PSMA-targeted ligand, ^175^Lu-DOTA-PSMA-GUL, including excretion and tissue distribution, are characterized for the first time by applying LC–MS/MS. The present data indicate that ^175^Lu-DOTA-PSMA-GUL exhibited linear pharmacokinetics over the dose range from 1 to 5 mg/kg and renal excretion is the primary elimination route. The prostate-specific targeting property of ^175^Lu-DOTA-PSMA-GUL was also demonstrated. The developed LC–MS/MS assay and the in vivo pharmacokinetic data would provide useful information for the further development of ^177^Lu-DOTA-PSMA-GUL and other structural analogs as a novel therapeutic agent for the treatment of prostate cancer.

## Supplementary Information

Supplementary Information.

## Data Availability

The datasets generated and analyzed during this study are available from the corresponding author upon reasonable request.
