# Supplementary material for: Determination of pharmacokinetics and tissue distribution of a novel lutetium-labeled PSMA-targeted ligand, 177Lu-DOTA-PSMA-GUL, in rats by using LC–MS/MS
Source: Sci Rep. 2022 Sep 14;12:15452. doi: 10.1038/s41598-022-19700-9 (PMC9474474; doi:10.1038/s41598-022-19700-9)
Supplement: Supplementary file 1 — Supplementary Information. [file 41598_2022_19700_MOESM1_ESM.pdf]

Supplementary Table 1. LC-MS/MS condition of ^{175}Lu-DOTA-PSMA-GUL in rat biomatrices.

Parameter	^{175}Lu-DOTA-PSMA-GUL	NOTA-PSMA-GUL (Internal standard 1)	Esomeprazole (Internal standard 2)
Polarity	Positive	Positive	Positive
m/z	522.1 → 148.0	385.8 → 188.9	346.1 → 198.0
Dwell time (ms)	100	100	100
Fragmentor (V)	380	380	380
Collision energy (V)	20	12	9
Gas temperature (°C)	220		
Gas flow (L/min)	14		
Nebulizer gas (psi)	40		
Sheath Gas Temperature (°C)	250		
Sheath Gas Flow (L/min)	11		
Capillary (V)	3000		
Nozzle Voltage (V)	1500		

Supplementary Table 2. Intra- and inter-day accuracy and precision of LLOQ and QC samples for ^{175}Lu-DOTA-PSMA-GUL in rat plasma, urine, feces, and 12 tissues.

Matrix	Concentration (ng/mL)	Intra-day (n=5)		Inter-day (n=5)	
		Accuracy (%)	Precision (%)	Accuracy (%)	Precision (%)
Plasma	20	96.06	4.03	98.16	5.11
	80	93.18	1.88	96.63	5.78
	8,000	92.56	2.11	98.15	7.64
	16,000	94.15	1.76	101.20	6.78
Urine	100	100.68	3.09	102.29	3.33
	400	95.64	2.91	98.65	5.89
	8,000	101.51	1.71	103.30	2.99
	16,000	102.87	1.88	104.38	5.05
Feces	100	101.46	4.01	99.57	1.87
	400	104.55	3.86	101.08	5.79
	1,600	104.39	3.51	100.46	4.13
	4,000	105.70	3.48	103.92	2.76
Brain	100	96.65	8.01	102.47	7.84
	400	93.64	5.38	93.75	3.12
	1,600	93.12	3.09	92.00	2.02
	4,000	94.74	4.94	93.58	2.76
Lung	100	103.28	2.57	102.08	4.96
	400	98.73	3.35	95.23	2.37
	1,600	95.95	2.39	98.32	8.32
	4,000	101.29	3.09	100.27	2.00
Heart	100	99.47	6.79	98.71	8.11
	400	100.13	3.91	94.55	2.18
	1,600	94.48	2.50	95.20	5.16
	4,000	96.14	5.69	98.37	4.49
Spleen	100	106.00	6.10	104.85	7.06
	400	101.98	6.16	97.89	5.13
	1,600	104.28	0.93	100.86	5.82
	4,000	106.26	1.75	104.82	3.41
Small intestine	100	98.00	5.05	101.70	4.81
	400	100.94	6.39	97.97	3.06
	1,600	102.71	5.01	101.47	4.75
	4,000	106.06	1.08	105.89	3.29
Stomach	100	104.58	5.65	98.88	7.80
	400	98.49	6.51	102.53	2.88
	1,600	95.35	4.11	96.45	5.43

	4,000	94.53	2.78	95.72	3.80
Kidney	100	106.82	4.35	103.00	3.95
	400	104.30	2.97	94.53	6.17
	1,600	101.95	2.35	94.71	2.96
	4,000	95.09	2.66	99.75	3.71
Liver	100	103.87	4.75	103.71	7.15
	400	100.43	3.29	99.95	4.84
	1,600	95.20	4.47	101.48	2.22
	4,000	93.18	2.01	94.28	6.28
Fat	100	105.81	1.59	99.73	6.52
	400	94.09	1.60	93.88	2.23
	1,600	93.18	2.69	92.99	2.36
	4,000	96.80	4.51	98.14	5.81
Muscle	100	101.71	10.22	105.37	5.13
	400	104.19	5.04	97.76	4.10
	1,600	106.82	2.79	100.12	4.36
	4,000	101.43	5.91	98.65	5.15
Prostate	100	102.23	7.12	104.72	5.07
	400	97.66	5.15	97.34	5.51
	1,600	99.24	6.16	102.40	3.36
	4,000	103.48	4.19	104.32	1.99
Testis	100	90.96	2.75	98.09	9.22
	400	104.33	4.13	103.06	3.24
	1,600	105.73	1.70	105.11	2.93
	4,000	97.46	4.44	96.64	5.33

Supplementary Table 3. Stability (%) of ^{175}Lu-DOTA-PSMA-GUL in the rat plasma, urine, feces, and 12 tissues.

Matrix	Concentration (ng/mL)	Short-term (n=4)	Long-term (n=4)	Freeze-thaw (n=4)	Autosampler (n=4)
Plasma	80	106.18 ± 0.41	100.61 ± 2.11	102.92 ± 2.77	97.69 ± 3.09
	16,000	101.14 ± 2.43	99.20 ± 0.51	98.68 ± 1.21	102.82 ± 1.73
Urine	400	91.67 ± 2.58	98.36 ± 1.67	105.67 ± 4.65	94.03 ± 2.84
	16,000	106.46 ± 1.17	100.28 ± 1.39	92.41 ± 1.12	104.69 ± 2.03
Feces	400	90.69 ± 6.38	103.52 ± 1.25	104.54 ± 6.86	97.85 ± 3.82
	4,000	104.76 ± 2.40	101.50 ± 1.39	105.65 ± 2.20	104.03 ± 2.53
Brain	400	92.98 ± 2.86	94.77 ± 3.27	96.03 ± 3.73	96.57 ± 0.76
	4,000	98.51 ± 2.55	95.22 ± 2.73	102.05 ± 3.19	90.60 ± 1.22
Lung	400	98.55 ± 4.32	99.08 ± 2.59	96.40 ± 1.10	98.84 ± 7.51
	4,000	96.87 ± 1.97	95.79 ± 1.75	97.55 ± 0.70	102.00 ± 4.06
Heart	400	95.08 ± 10.83	93.97 ± 1.45	95.08 ± 10.83	98.75 ± 4.29
	4,000	91.74 ± 0.93	93.50 ± 2.78	97.19 ± 8.21	92.56 ± 3.26
Spleen	400	101.61 ± 2.41	97.10 ± 3.45	98.46 ± 3.74	102.59 ± 1.80
	4,000	104.24 ± 4.36	106.40 ± 3.85	104.53 ± 2.39	102.93 ± 2.02
Small intestine	400	99.17 ± 2.35	98.80 ± 2.76	99.15 ± 6.00	98.68 ± 2.52
	4,000	102.12 ± 2.51	103.65 ± 4.10	100.54 ± 2.33	104.13 ± 2.24
Stomach	400	98.96 ± 4.28	99.72 ± 2.41	97.59 ± 1.68	100.00 ± 4.55
	4,000	96.98 ± 3.91	94.79 ± 3.41	92.47 ± 1.38	93.61 ± 2.79
Kidney	400	95.45 ± 3.51	95.92 ± 2.97	94.83 ± 2.49	95.72 ± 4.19
	4,000	98.14 ± 3.63	94.61 ± 2.50	94.30 ± 1.02	97.98 ± 2.82
Liver	400	98.94 ± 5.66	97.86 ± 1.87	95.93 ± 3.91	97.39 ± 3.47
	4,000	91.39 ± 2.47	91.83 ± 1.56	91.72 ± 2.23	92.47 ± 2.32
Fat	400	98.86 ± 3.38	96.21 ± 4.47	91.54 ± 1.36	95.57 ± 4.47
	4,000	96.74 ± 3.52	94.41 ± 3.10	99.64 ± 2.85	95.05 ± 0.70
Muscle	400	100.99 ± 2.76	99.56 ± 2.47	101.47 ± 2.25	94.93 ± 2.73
	4,000	99.13 ± 2.50	99.74 ± 4.41	94.67 ± 0.44	98.62 ± 2.06
Prostate	400	95.76 ± 1.48	95.50 ± 3.57	95.17 ± 3.44	97.82 ± 2.46
	4,000	99.30 ± 3.24	97.12 ± 3.90	99.85 ± 2.74	96.30 ± 0.83
Testis	400	100.51 ± 3.98	103.74 ± 3.80	107.18 ± 0.85	101.68 ± 6.54
	4,000	101.72 ± 1.48	94.96 ± 4.89	99.52 ± 0.96	97.56 ± 4.96

Supplementary Table 4. Process efficiency (%) of ^{175}Lu-DOTA-PSMA-GUL and its internal standards in blank rat plasma, urine, feces, and 12 tissues (n=4).

Matrix	^{175}Lu-DOTA-PSMA-GUL		Internal standard*	
	Low QC (n=4)	High QC (n=4)	Low QC (n=4)	High QC (n=4)
Plasma	98.66 ± 4.85	99.40 ± 1.95	84.86 ± 5.00	86.04 ± 4.29
Urine	77.29 ± 9.66	76.83 ± 0.99	93.83 ± 6.79	95.31 ± 0.79
Feces	77.51 ± 5.12	75.96 ± 2.30	97.74 ± 3.42	103.97 ± 6.08
Brain	69.31 ± 3.85	69.50 ± 4.79	97.19 ± 3.09	102.58 ± 9.47
Lung	21.80 ± 0.53	23.41 ± 0.37	97.49 ± 1.52	103.20 ± 5.18
Heart	51.88 ± 1.33	51.29 ± 3.34	95.87 ± 2.34	98.69 ± 9.98
Spleen	53.94 ± 5.34	50.63 ± 3.24	97.86 ± 4.99	100.22 ± 9.05
Small intestine	30.97 ± 2.48	28.57 ± 0.76	99.35 ± 6.44	103.35 ± 10.65
Stomach	62.84 ± 3.63	60.66 ± 2.28	97.14 ± 4.40	107.98 ± 6.70
Kidney	18.66 ± 1.23	17.98 ± 0.69	118.82 ± 0.53	110.61 ± 1.34
Liver	15.17 ± 0.57	13.01 ± 0.61	101.22 ± 2.75	108.59 ± 9.97
Fat	99.37 ± 6.30	100.64 ± 3.58	84.38 ± 1.83	82.22 ± 1.63
Muscle	101.50 ± 4.15	99.76 ± 6.17	91.97 ± 3.56	102.31 ± 9.81
Prostate	42.24 ± 0.85	40.48 ± 1.11	93.57 ± 3.54	98.81 ± 5.94
Testis	122.64 ± 5.12	124.06 ± 6.85	98.38 ± 3.08	104.26 ± 6.44

*, NOTA-PSMA-GUL (IS1) for plasma, and esomeprazole (IS2) for urine, feces, and tissues.

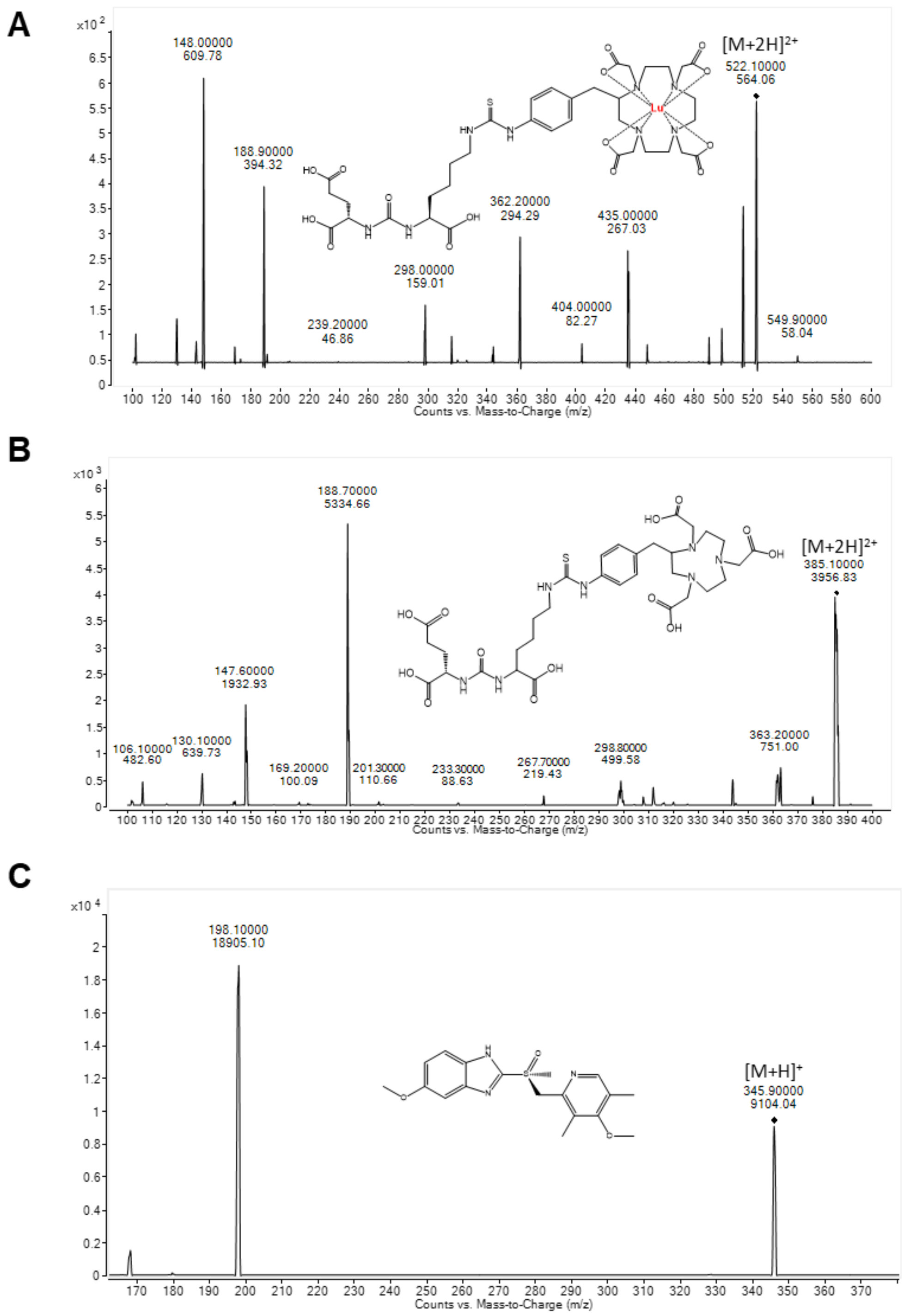

Supplementary Figure 1. Product ion mass spectra of protonated (A) ^{175}Lu-DOTA-PSMA-GUL (522.1 → 148), (B) NOTA-PSMA-GUL (385.8 → 188.9), and (C) esomeprazole (346.1 → 198) in positive ionization mode.

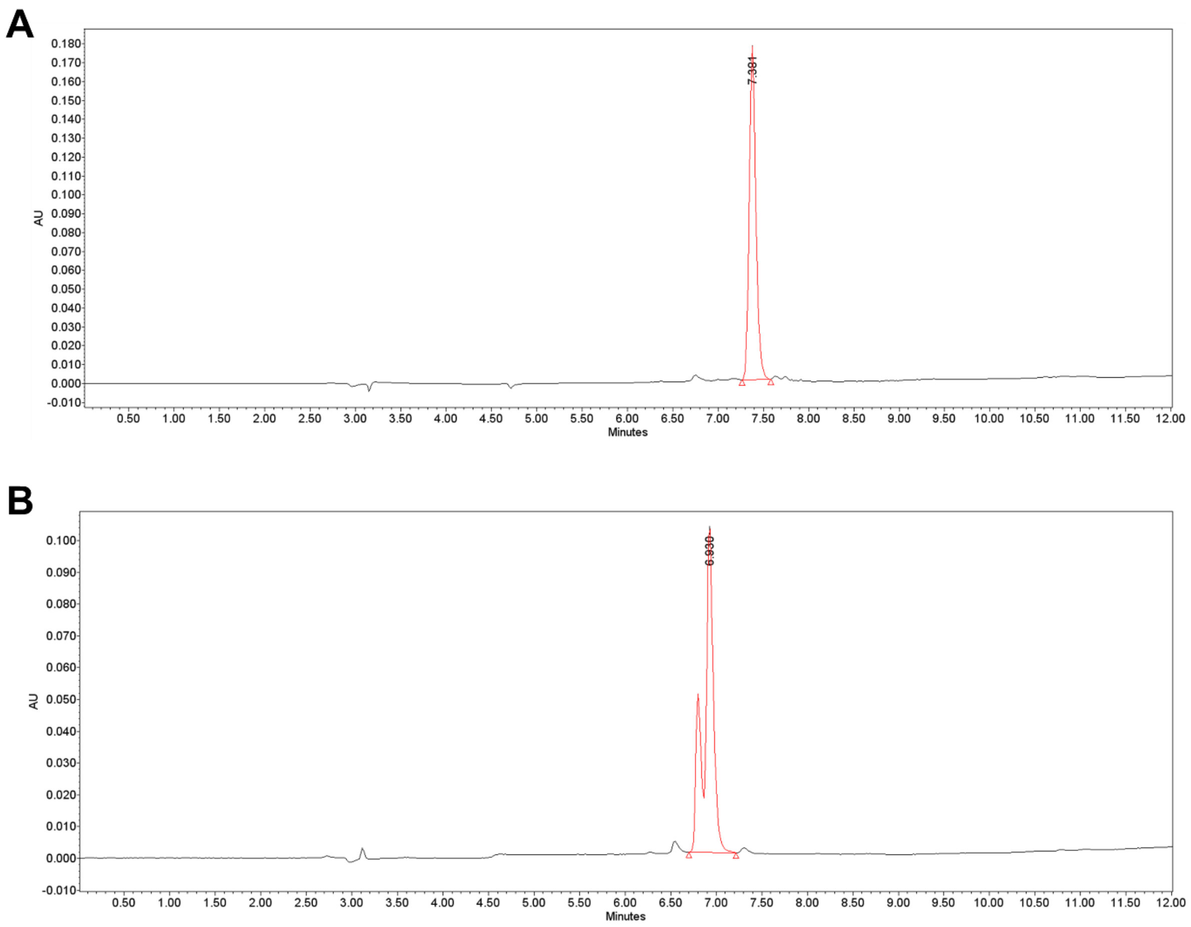

Supplementary Figure 2. The HPLC-UV chromatograms of (A) DOTA-PSMA-GUL and (B) ^{175}Lu-DOTA-PSMA-GUL.
